# Differential Lesion Patterns Associated With Stroke‐Induced Apraxia in Women and Men

**DOI:** 10.1111/ene.70201

**Published:** 2025-05-23

**Authors:** Nina N. Kleineberg, Claudia C. Schmidt, Gereon R. Fink, Peter H. Weiss

**Affiliations:** ^1^ Department of Neurology Faculty of Medicine and University Hospital Cologne, University of Cologne Cologne Germany; ^2^ Cognitive Neuroscience Institute of Neurosciences and Medicine (INM‐3), Forschungszentrum Jülich Jülich Germany

**Keywords:** gender, imitation, pantomime, sex differences, voxel‐based lesion–symptom mapping (VLSM)

## Abstract

**Background:**

The motor‐cognitive syndrome apraxia is a common stroke sequela and severely affects the outcome after stroke by impairing activities of daily living. Notably, like in many health conditions, there is a massive backlog regarding studies on sex differences in patients with apraxia despite common knowledge that sex influences praxis performance in healthy participants. We investigated putative sex differences in apraxic stroke patients at the behavioral and neural levels.

**Methods:**

We retrospectively analysed the data of a cohort of 102 left‐hemisphere stroke patients in the (sub)acute phase who were apraxic according to the Cologne Apraxia Screening (KAS). We conducted voxel‐based lesion–symptom mapping (VLSM) to elucidate the lesion patterns. Further, in an age‐matched subsample (tolerance of 5 years) with equal numbers of men and women, behavioral comparisons and a VLSM analysis were conducted to explore differential sex‐related lesion patterns.

**Results:**

Overall, apraxic deficits were associated with lesions in the parietal, temporal, and frontal regions in the cohort of 102 left‐hemisphere stroke patients. The age‐matched cohort consisted of 30 women and 30 men and showed no significant differences in demographic and clinical characteristics. There were no performance differences between men and women at the behavioral level regarding praxis functions. In contrast, VLSM revealed differential lesion patterns by sex. Male compared to female apraxic stroke patients significantly more often showed lesions that affected the left inferior frontal gyrus.

**Conclusion:**

The data suggest a differential organization of the praxis system in men and women, warranting further exploration.

## 1 Introduction

Apraxia is a motor‐cognitive disorder leading to impaired performance of skilled or purposeful movements frequently observed after left‐hemisphere (LH) stroke. Apraxic deficits cannot be solely explained by basal sensorimotor, aphasic, or other cognitive stroke‐related deficits and affect both limbs. Apraxia manifests through heterogeneous impairments, including gesture imitation, pantomime of object use, and actual object use [1].

The individual burden of a stroke is modulated by nonmodifiable and modifiable factors. For instance, it has been shown that older females have a greater prevalence of and worse outcomes after ischemic stroke than males and younger females [2, 3]. Notably, age, sex, and education significantly impacted the performance in neuropsychological assessments [4]. For example, lower education levels and higher age significantly reduced gesture production skills in healthy elderly subjects [5]. Thus, biological (e.g., sex, age, and ethnic group) and sociodemographic (e.g., gender identity, education, and lifestyle) factors should receive more attention in studies. We report sex differences regarding the biological sex as assigned at birth, using a simplified binary variable termed as males/females and men/women. Sex differences have been described in neurodegenerative and neuropsychiatric diseases [3, 6] and various domains of cognitive neuroscience, such as the “stereotype” of a male advantage in spatial abilities and mental rotation tasks [7, 8], and a female advantage in verbal skills [9, 10], body language processing and social cognition [11]. Furthermore, sex modulated task‐dependent motor performance, such as a male advantage in targeted motor skills [12], and in maximum finger‐tapping frequency, and strength [13]. Moreover, men performed better in gross motor tasks, while females outperformed men in dexterous tasks [14]. However, some of the reported sex differences are not undisputed [15, 16, 17] considering that some of the so‐called “sex‐specific” differences may result from group differences that confound task‐related results, for instance, by physical differences and psychosocial factors such as gender identity, sexual orientation, as well as experience and training leading to culturally framed “gender stereotypes” [17, 18]. Additionally, in patient studies, many further factors, for example, etiologies and comorbidities, may differ between men and women and possibly contribute to sex differences in task performance.

More recently, sex differences at the neurobiological level that could account for the behavioral differences were observed. Differences in cortical thickness in specific brain regions of men versus women were related to a female advantage in verbal memory and a male advantage in visuospatial memory [19]. Also, sex differences in brain network connectivity were revealed during mental rotation performance [20].

So far, only few studies have assessed the effects of sex on praxis. Girls performed better in gesture imitation than boys at preschool [21]. In adulthood, women conducted a serial production task of learned gestures significantly better than men [22], and women were faster in repeating a sequence of movements [23]. The female advantage in gesture imitation was still found in elderly community‐dwelling people and patients with mild cognitive impairment or dementia [24]. In contrast, another study investigating gesture production in healthy elderly participants found no sex differences [5]. When pantomimed reach‐to‐grasp actions were compared to actual object grasping, men and women showed no relevant kinematic differences between pantomimed and actual grasping. However, with visual feedback, the performance of the pantomimed grasp assimilated to the actual object grasp kinematics in men but not in women, showing a sex‐related difference in the presence of visual feedback [25]. Observing gestures toward the body (compared to away from the body) led to a greater pupil dilation in females than in males, which was interpreted as women being more attuned and responsive to social cues [26].

The wealth of studies investigating apraxia contrasts with the dearth of studies examining sex differences in patients with apraxia. In an early study, Kimura observed potential sex differences in language and praxis deficits after stroke or tumor, suggesting that language and praxis functions may be organized differently in the brains of men and women [27].

Accordingly, we aimed to systematically investigate sex differences in apraxia and the underlying lesion correlates in LH stroke patients. We analysed a sample of 102 patients with LH ischemic stroke suffering from apraxia according to the Cologne Apraxia Screening (KAS). To investigate sex differences, we conducted a case–control matching for the factor age to minimize the potentially confounding effect of age on stroke severity and praxis performance [5, 28]. In the resulting age‐matched sample of 60 apraxic LH stroke patients, we compared apraxic deficits on a behavioral level. We performed voxel‐based lesion–symptom mapping (VLSM) to unravel potential differential lesion patterns associated with apraxia in male and female LH stroke patients.

## 2 Material and Methods

### 2.1 Patient Sample

We retrospectively analysed behavioral and lesion data in 102 patients after their first unilateral, ischemic LH stroke suffering from apraxia according to the KAS [29]. The sample was extracted from the cohort of Kleineberg and colleagues [30] consisting of 194 stroke patients. The remaining 92 patients in that study did not suffer from apraxia. For all 102 patients, apraxia assessments and a lesion map suitable for statistical lesion analysis were available. All included patients had given written informed consent during their participation in the original studies that all their data may be used for further analyses and studies, like the current retrospective study. This procedure was approved by the Ethics committee of the Medical Faculty of the University of Cologne.

All patients were right‐handed according to the Edinburgh Handedness Inventory [31] and tested in the (sub)acute phase (i.e., within 3 months) after stroke.

### 2.2 Neuropsychological Assessment

Apraxic deficits were assessed by the KAS [29], a validated and standardized apraxia assessment comprising four subtests: two on pantomime of object use and two on gesture imitation. In the pantomime tests, the patients are shown pictures of objects and asked to demonstrate the typical use with their left (ipsilesional) arm/hand. The first pantomime test includes bucco‐facial movements, while the second part draws upon the upper limb as the only effector. In the imitation tasks, photographs of a woman performing gestures are shown, and the patients are asked to replicate the gesture. One imitation subtest consists of bucco‐facial gestures, that is, the face/mouth as effector, and the other of arm/hand gestures. In all subtests, a maximum of four points per item/gesture can be reached. The total sum score of the KAS is 80 points, with a cut‐off value for apraxia of ≤ 76 points.

Aphasia was assessed using the short version of the aphasia checklist (ACL‐K) [32], which consists of a reading‐aloud task, a verbal speech comprehension task, a word generation task, and a rating of the verbal fluency by the examiner. A score less than 33 points indicates aphasia.

### 2.3 Statistical Analyses of the Descriptive and Behavioral Data

Data analyses were performed using IBM SPSS Statistics (Statistical Package for the Social Sciences, version 24, SPSS Inc. Chicago Illinois, USA). To compare sex differences in apraxic deficits in the 102 apraxic stroke patients that comprised 33 women and 69 men, we performed a case–control matching for age using sex as the group indicator. We matched with a tolerance of 5 years, leaving three cases without a match, resulting in an age‐matched cohort of 60 patients (30 women, 30 men). We investigated differences between men and women in descriptive, clinical, and neuropsychological data by Mann–Whitney U test. To compare the stroke territories, we manually screened the lesion maps of all patients and identified the main territory of stroke. We extracted the number of lesioned voxels with the MRIcron software package (https://www.nitrc.org/projects/mricron).

In the age‐matched cohort, we conducted a repeated measures ANOVA with the within‐subject factors DOMAIN (pantomime vs. imitation) and EFFECTOR (bucco‐facial vs. arm/hand) using SEX as a between‐subject factor.

For further interpretation of the sex‐specific analyses in LH stroke patients, we analysed the KAS performance in a healthy control group and generated z‐transformed KAS scores of the stroke patients based on the KAS performance of the age‐ and sex‐matched healthy control participants (see Suppl. Section A).

### 2.4 Lesion Delineation and Analyses of Lesion Data

Lesion mapping was conducted manually using the patients' clinical MRI (*n* = 70) or CT (*n* = 32) scans. The lesions were delineated in an axial slice distance of 5 mm in MNI space of a T1‐weighted template MRI scan (ch2.nii) from the Montreal Neurological Institute (MNI) with a 1 mm x 1 mm resolution that best matched the individual slices of each patient's scan. Two experienced investigators, blind to the clinical data, had to agree on the lesion locations and extent jointly.

VLSM analyses were conducted using the nonparametric mapping (NPM) program distributed with MRIcron. First, we conducted a VLSM analysis with all patients (*n* = 102) to identify the associated lesion patterns for the patients' apraxic deficits at the voxel level [33, 34]. Voxel‐wise t‐tests were calculated to compare the continuous behavioral scores (KAS score) of the patients with and without a lesion in each voxel. Only voxels lesioned in at least 10% of the patients were considered. Voxels were considered significant at a statistical threshold of *p* < 0.05, corrected for multiple comparisons with the false discovery rate (FDR).

Second, we investigated potential sex differences in lesion patterns associated with apraxia in the age‐matched cohort. We applied the voxel‐wise Liebermeister Test for the binary variable sex [34], with an FDR‐corrected threshold of *p* < 0.05. For all VLSM results, clusters with the respective peak voxel's Z‐values were extracted using MRIcron. The automated anatomical labeling (AAL) atlas determined the clusters' anatomical location. All significant lesioned voxels are shown in the figures, but only clusters with at least 20 voxels are reported and further interpreted, similar to Schmidt and colleagues [35]. As exploratory analyses, we investigated the interrelation of damage to the lesion sites revealed by the VLSM of male versus female stroke patients with apraxic deficits (see Suppl. Section B).

## 3 Results

### 3.1 Clinical and Behavioral Data

The initial cohort of 102 apraxic patients after LH stroke consisted of 69 men and 33 women with a mean (±SD) age of 65.7 ± 13.1 years. They were assessed at 22.8 ± 17.8 days post‐stroke. According to the inclusion criteria, all patients were apraxic based on the KAS with a mean score of 60.9 ± 15.1 points. Of the 102 LH stroke patients, 86 were aphasic as assessed with the ACL‐K (mean score 19.6 ± 11.6 points).

Table 1 provides descriptive data for the age‐matched cohort. Notably, there were no significant differences between male and female patients in demographic and clinical characteristics, time post‐stroke, stroke territory, lesion size, aphasia severity, and education level.

**TABLE 1:** Demographic and clinical data of the age‐matched left‐hemisphere stroke cohort with apraxia, and of the female and male patients.

	Age‐matched cohort *N* = 60	Women *N* = 30	Men *N* = 30	*p*
**Demographic and clinical data**				
Age (years)	68.2 ± 13.5, 72 [25], 43–87	67.8 ± 13.7, 73 [44], 43–87	68.6 ± 13.6, 71 [24], 44–87	0.662
Laterality quotient	*N* = 53 92.0 ± 13.1, 100 [14.1], 40–100	*N* = 29 90.2 ± 15.5, 100 [20], 40–100	*N* = 24 94.3 ± 9.0, 100 [11], 70–100	0.406
Education (years)	*N* = 43 13.1 ± 2.7, 13 [0], 8–21	*N* = 23 12.5 ± 2.4, 13 [3], 8–18	*N* = 20 13.9 ± 3.0, 13 [2], 8–21	0.083
Time post‐stroke (days)	20.6 ± 16.6 16.5 [25], 1–76	21.5 ± 16.9 21.4 [26], 2–76	19.7 ± 16.6 14.5 [26], 1–63	0.723
Scan used for lesion mapping MRI CT	42 (70.0%) 18 (30.0%)	22 (73.3%) 8 (27.7%)	20 (66.7%) 10 (33.3%)	0.573
Main stroke territory MCA PCA ACA	49 (81.7%) 9 (15.0%) 2 (3.3%)	24 (80.0%) 6 (20.0%) 0 (0.0%)	25 (83.3%) 3 (10.0%) 2 (6.7%)	0.221
MCA territory involved	53 (88.3%)	26 (86.7%)	27 (90.0%)	0.688
PCA territory involved	15 (25.0%)	9 (30.0%)	6 (20.0%)	0.371
ACA territory involved	3 (5.0%)	1 (3.3%)	2 (6.7%)	1.0
Lesion size (number of voxels lesioned)	8620 ± 12329, 4329 [8716], 30–60565	8291 ± 12195, 4197 [8700], 30–60565	8949 ± 12662, 4329 [9660], 299–46900	0.976
Patients with aphasia (by ACL‐K)	48 (81.4%)	25 (83.3%)	23 (79.3%)	0.692
ACL‐K Sum score	*N* = 59 21.6 ± 11.1, 24.5 [19.5], 1.5–38	*N* = 30 23.1 ± 9.5, 25 [15.4], 3.5–38	*N* = 29 20.0 ± 12.6, 22 [22], 1.5–38	0.387

*Note:* Values are shown as mean ± SD (standard deviation), median [interquartile range], min–max. *p* values are derived from the Mann–Whitney U test, chi‐square test, or Fisher's exact test.

Abbreviations: ACL‐K, short version of the aphasia‐check list; ACA, anterior cerebral artery; MCA, middle cerebral artery; PCA, posterior cerebral artery.

The 2 × 2 rm‐ANOVA on the KAS scores with the within‐subject factors DOMAIN (pantomime vs. imitation) and EFFECTOR (bucco‐facial vs. arm/hand) and between‐subject factor SEX revealed no significant main effect of SEX [F_(1,58)_ = 0.50, *p* = 0.482]. Furthermore, the two‐way interactions DOMAIN × SEX [F_(1,58)_ = 0.82, *p* = 0.370] and EFFECTOR × SEX [F_(1,58)_ = 0.03, *p* = 0.867] and the three‐way interaction of DOMAIN x EFFECTOR × SEX [F_(1,58)_ = 0.55, *p* = 0.455] were not significant. The scores for the domains and effectors of the KAS are shown in Table 2.

**TABLE 2:** Results of the apraxia assessments in the age‐matched left‐hemisphere stroke cohort, and in the female and male patients.

	Age‐matched cohort *N* = 60	Women *N* = 30	Men *N* = 30	*p*
*KAS total sum*	60.1 ± 17.1, 65 [65], 0–76	61.7 ± 15.3, 66 [14], 8–76	58.5 ± 18.8, 62 [19], 0–76	0.549
KAS pantomime subtests	30.2 ± 10.2, 34 [11.8], 0–40	30.5 ± 9.9, 34 [8], 0–40	29.9 ± 10.6, 32 [14.5], 0–40	0.988
KAS imitation subtests	29.9 ± 8.7, 33 [10], 0–40	31.1 ± 7.4, 34 [8], 8–40	28.6 ± 9.8, 32 [10], 0–40	0.304
KAS bucco‐facial subtests	32.8 ± 8.9, 36 [7.5], 0–40	33.5 ± 7.5, 36 [6.5], 4–40	32.1 ± 10.2, 35.5 [10], 0–40	0.853
KAS arm/hand subtests	27.3 ± 9.7, 29.5 [14], 0–40	28.2 ± 9.4, 32 [12.5], 4–40	26.4 ± 10.2, 27.5 [16.3], 0–40	0.448

*Note:* Values are shown as mean ± SD (standard deviation), median [interquartile range], min–max. *p* values are calculated by Mann–Whitney U tests.

Abbreviation: KAS, Cologne Apraxia Screening.

Summing up, at the behavioral level, there were no statistically significant sex differences regarding the investigated apraxic deficits in the age‐matched apraxic LH stroke patients.

Note that also the z‐transformed KAS scores showed no significant differences in apraxic deficits between female and male stroke patients with apraxia (see Table S2).

### 3.2 Lesion Correlates of Apraxia and Differential Sex‐related Lesion Patterns

**FIGURE 1:**
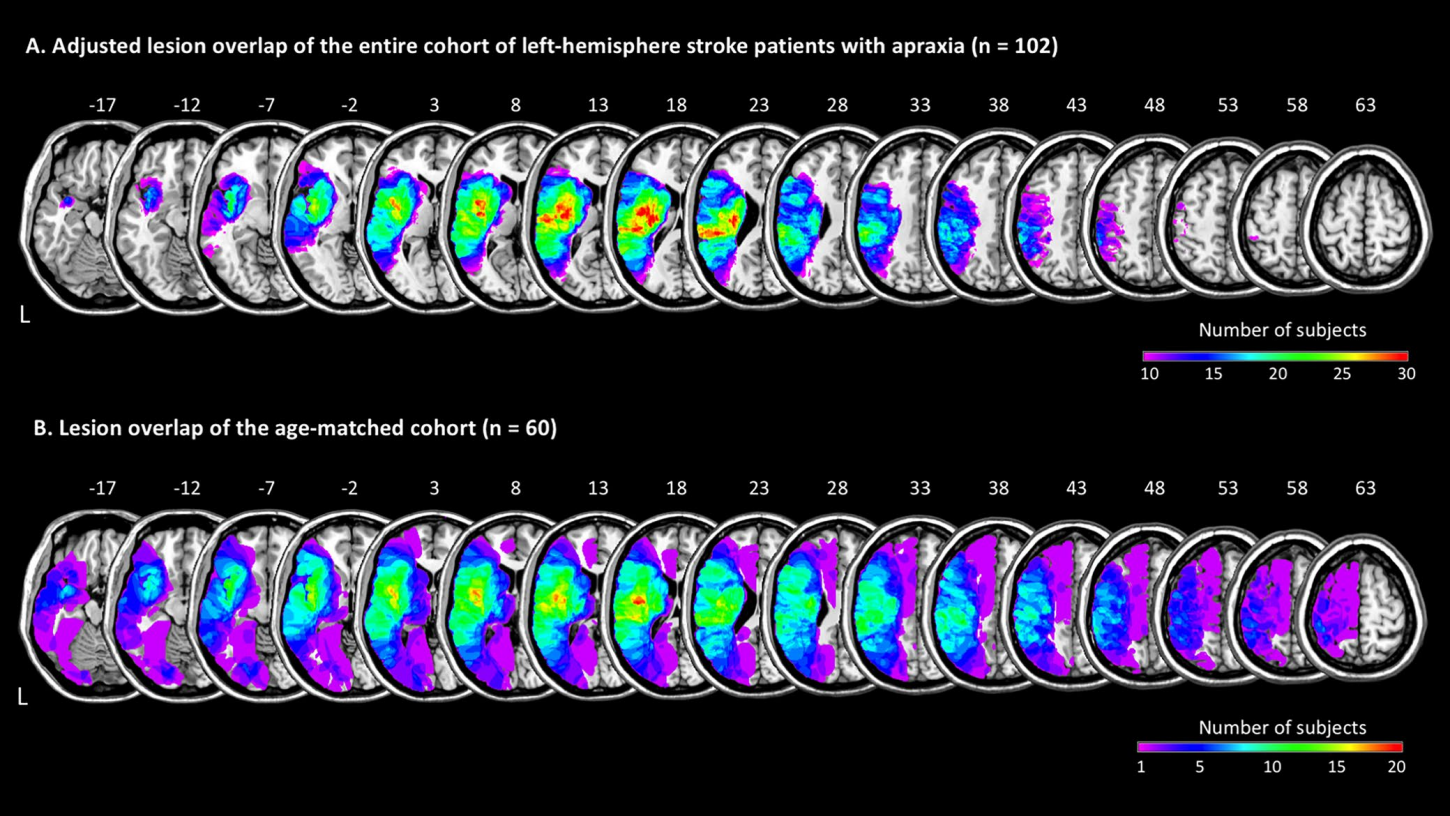
Lesion overlaps. (A) Adjusted lesion overlap of the 102 patients with apraxia after left‐hemisphere stroke. Only voxels lesioned in at least 10% of the patients are shown. (B) Nonadjusted lesion overlap of the age‐matched cohort with equal sex distribution (30 women, 30 men). Color shades represent the increasing number of overlapping lesions. Axial slices with MNI z‐coordinates from ‐17 to +63 are shown.

The lesion overlaps of the initial cohort of LH stroke patients with apraxia and the age‐matched subsample revealed very similar lesion distributions, with the main overlap in the insular region, rolandic operculum, superior temporal gyrus (STG), postcentral gyrus, supramarginal gyrus (SMG), and inferior frontal gyrus (IFG, see Figure 1).

**FIGURE 2:**
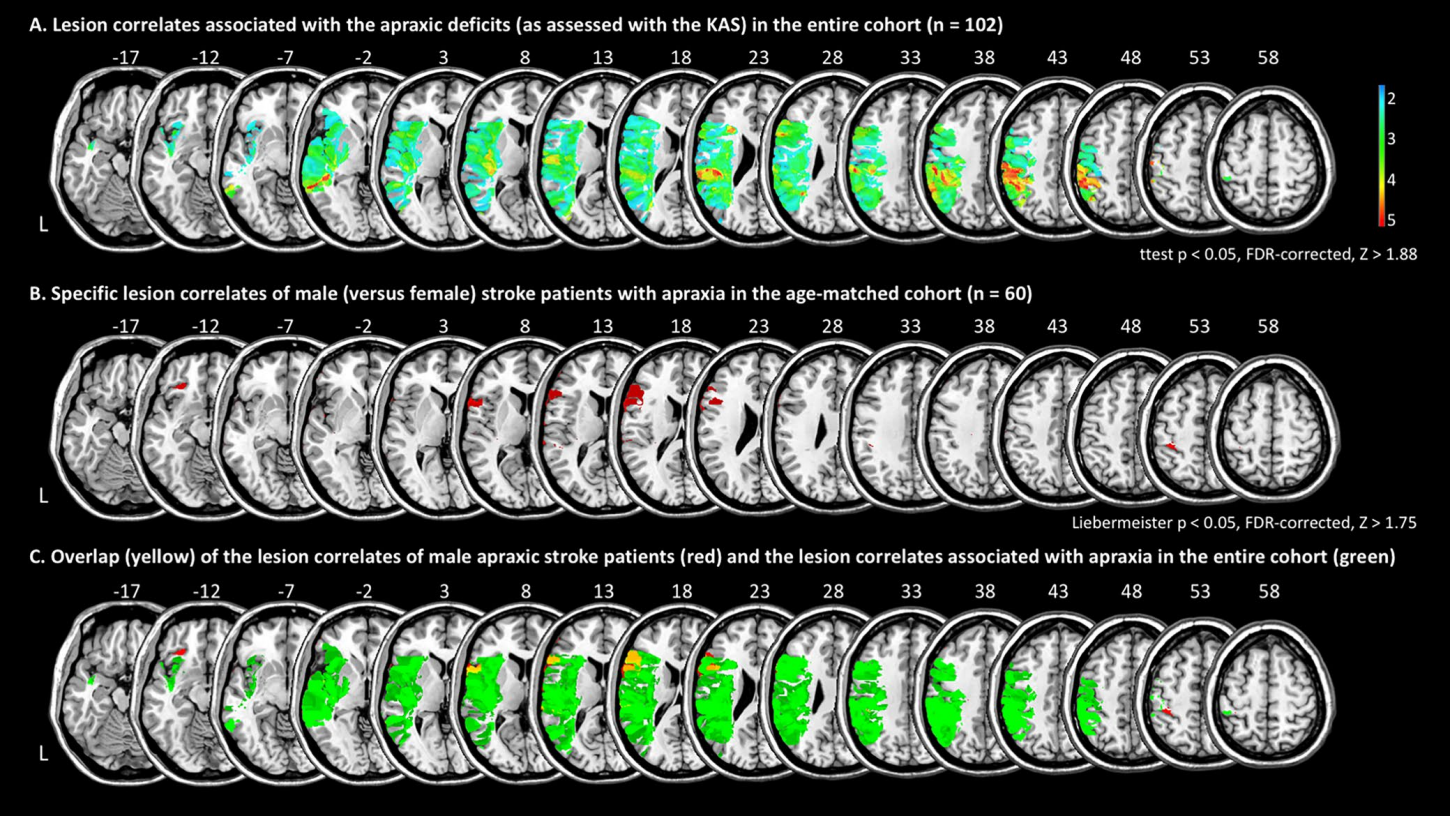
Results of the statistical lesion analyses. (A) Results of the VLSM analysis: Lesion correlates are shown that were significantly associated with poorer performance in the Cologne Apraxia Screening (KAS), that is, with more severe apraxic deficits in the sample of 102 apraxic patients after left‐hemisphere stroke. Only voxels lesioned in at least 10% of the patients were included in the VLSM analysis. Voxels are thresholded at *p* < 0.05 (FDR‐corrected, *t*‐test). (B) Lesion correlates of male versus female apraxic stroke patients in the age‐matched cohort with equal sex distribution. All voxels were included in this analysis, and voxels are thresholded at *p* < 0.05 (FDR‐corrected, Liebermeister test). (C) Overlap of the specific lesion correlates for men versus women with apraxia (*2B, here in red*) with the lesion correlates associated with apraxic deficits (*2A, here in green*) in the 102 LH stroke patients. The overlap is shown in *yellow*. Axial slices with MNI z‐coordinates from −17 to +58 are shown.

For the cohort of 102 LH stroke patients, VSLM revealed the lesion correlates associated with apraxia (Figure 2A). Please see Table 3 for the corresponding MNI coordinates of the peak voxels of the clusters and the respective (maximum) Z‐values according to the association strength. The IPL (including the SMG) showed the strongest lesion association with poorer performance in the KAS.

**TABLE 3:** Lesion correlates associated with the apraxia score of the KAS (Cologne Apraxia Screening) in the entire cohort (*n* = 102, as assessed with VLSM).

Brain region	Maximum *Z*‐value	Peak MNI coordinates x, y, z	Cluster size (voxels)
Inferior parietal lobe (IPL)	5.95	−45, −30, +43	1725
Supramarginal gyrus (SMG)	5.62	−56, −27, +38	1918
Angular gyrus (AG)	3.78	−40, −66, +43	88
Superior temporal gyrus (STG)	4.51	−59, −11, +13	2925
Middle temporal gyrus (MTG)	5.32	−64, −45, −2	2696
Middle frontal gyrus	4.13	−53, +15, +38	537
Inferior frontal gyrus (IFG)	2.76	−61, +14, +13	93
Rolandic operculum	5.25	−44, −30, +23	3191
Insula	3.71	−38, +16, −12	270
Precentral gyrus	4.26	−62, +9, +33	589

*Note:* For each brain region (minimum cluster size of 20 voxels), the maximum *Z‐*value and the MNI coordinates of the corresponding peak voxel are reported. All lesion correlates are significant at *p* < 0.05, FDR corrected.

Since there were no significant sex differences in apraxic deficits at the behavioral level, we conducted a VLSM analysis comparing lesions of male versus female apraxic LH stroke patients. Interestingly, the IFG was significantly more affected in male than in female apraxic patients (Figure 2B and Table 4). No significant lesion correlates were found for the opposite comparison, that is, when apraxic female LH stroke patients were compared to males. Overlaying these sex‐specific clusters of male versus female apraxic stroke patients with the lesion clusters that were associated with a poorer KAS score in the stem cohort of 102 LH stroke patients revealed that the main lesion correlates of male LH stroke patients with apraxia in the IFG were located within the lesion network accounting for apraxic deficits (see overlay, yellow regions in Figure 2C).

**TABLE 4:** Differential lesion correlates of male versus female left‐hemisphere stroke patients with apraxia in the age‐matched cohort (*n* = 60, as assessed with VLSM).

Brain region	Maximum *Z*‐value	Peak MNI coordinates *x*, *y*, *z*	Cluster size (voxels)
IFG, pars opercularis	2.83	−62, +17, +18	352
IFG, pars orbitalis	1.94	−32, +29, −12	59
IFG, pars triangularis	2.26	−56, +28, +23	34
Postcentral gyrus	2.26	−35, −36, +53	42

*Note:* For each brain region (minimum cluster size of 20 voxels), the maximum Z‐value and the MNI coordinates of the corresponding peak voxel are reported. All lesion correlates are significant at *p* < 0.05, FDR corrected.

Abbreviation: IFG, inferior frontal gyrus.

For results of the exploratory analyses between the LH apraxic stroke patients, whose lesions also comprised the differential sex‐specific lesion sites, versus those who did not, and the significant effect on the KAS score, see Suppl. Section B.

## 4 Discussion

Data revealed no significant differences regarding apraxic deficits between men and women at the behavioral level. However, male LH stroke patients with apraxia (relative to females) showed differential lesion correlates in the left IFG that were mainly located within the known praxis network of the left hemisphere.

Notably, these differential lesion correlates could not be explained by differences at the behavioral level but instead represented sex differences in the lesion patterns underlying apraxic deficits in LH stroke patients. The relevance of these lesion sites for praxis performance was reflected by a significant decrease in the KAS score in the patients with lesions encompassing the sex‐specific lesion sites for apraxia. This decrease was even more pronounced in male apraxic stroke patients. This exploratory analysis (reported in Suppl. Section B) corroborated our findings of sex‐related differential lesion patterns and their influence on apraxic deficits.

### 4.1 Lesions Associated With Apraxia

Confirming previous findings, the current apraxia‐related lesion correlates, as revealed by VLSM in the 102 apraxic LH stroke patients, comprised the known left‐lateralized praxis networks, involving the parietal, temporal, and frontal regions [36]. In this network, the inferior parietal lobe (IPL) represents a key hub, recently again confirmed in a large anatomical likelihood estimation meta‐analysis of lesion–symptom mapping studies [37]. In our data, the IPL's importance in apraxia is reflected in its Z‐value, which holds the highest association strength with poor KAS performance. Besides the parietal lesions, all other lesion correlates associated with apraxic deficits in our VLSM analysis replicated findings of previous studies, such as the involvement of the middle temporal gyrus, STG, insular region, precentral areas, and the IFG [38, 39, 40]. Thus, our cohort of 102 apraxic LH stroke patients, serving as a stem cohort for the sex‐related investigations, showed a typical lesion pattern associated with apraxia.

### 4.2 Apraxic Deficits of Female and Male Left‐hemisphere Stroke Patients

There were no significant sex differences in apraxic deficits at the behavioral level. Healthy control data supported these results (see Suppl. Section A). Thus, previously reported advantages of (healthy) women in gesture imitation and production [21, 22, 24] were not evident in the KAS scores of a healthy control group nor the apraxic stroke patients. This finding aligns with previous studies that did not observe sex‐related differences in gesture production in healthy elderly subjects [5].

### 4.3 Differential Lesion Correlates in Apraxic Men and Women

As there was no difference in apraxic deficits at the behavioral level, we could directly compare the lesion patterns between apraxic men and women with LH stroke. Apraxic men, relative to women, showed a differential involvement of lesions in the left IFG.

To allow such inference, it was essential to consider and rule out the following potential confounding factors. Since women are known to suffer from more severe strokes with worse prognosis, attributed to older age [28], and since motor and praxis performances decline with age [5, 13], age‐matching was important to investigate sex differences in apraxic deficits.

Furthermore, group comparisons ruled out a relevant difference in stroke size and main stroke territory between men and women. Of note is that the here reported VLSM results were obtained without correction for lesion size. When regressing out lesion size in the VLSM analysis on sex differences, no statistically significant clusters remained after correction for multiple comparisons. However, uncorrected, similar lesion patterns to the here reported ones evolved, pointing to the effects of reduced power [41]. Aside from that, a generally higher lesion load in the IFG in men compared to women was not expected based on clinical data on main lesion sites after stroke. Frontal areas are more often affected in women after an infarction in the territory of the middle cerebral artery (MCA). In contrast, men exhibit significantly more often stroke lesions in parieto‐temporal areas after MCA stroke [42]. Thus, the differential lesion pattern in the IFG of male compared to female apraxic LH stroke patients cannot be explained by unspecific sex‐related differences in the distribution of stroke lesions. In turn, they rather represent specific sex differences in the lesion patterns underlying apraxic deficits, suggesting a differential organization of the praxis system between men and women.

A differential organization of the praxis system was first suggested more than 40 years ago [27]. This early study reported descriptively a lower mean score for “manual praxis” in female compared to male patients suffering from stroke or tumors. The authors found a tendency that “manual apraxia” in women compared to men occurred more often from damage to the anterior part of the LH than from posterior LH damage (divided by the Rolandic fissure). However, this finding was based on qualitative lesion descriptions without statistical lesion–symptom mapping, limiting the interpretation and generalizability of their findings.

The current study adopting statistical lesion–symptom mapping revealed a sex‐specific lesion correlate for apraxia in the left IFG of male stroke patients. As part of the praxis network, lesions to the left IFG were shown to be specifically associated with pantomime [38, 43] and gesture imitation deficits in mixed samples of female and male LH stroke patients [40, 44, 45].

Our data suggest that men who suffer from a lesion to the left IFG exhibit apraxic deficits rather than women. This difference could point to a more distributed organization of the praxis system in women with a potentially more bilateral representation of IFG‐related praxis functions. Such a distributed praxis system would enable women to better compensate for unilateral lesions to the critical nodes of the LH praxis networks (e.g., left IFG). Of note, a more distributed praxis system in women could also explain why the current VLSM analysis did not reveal specific lesion correlates of apraxic deficits in female LH stroke patients with apraxia when compared to males. The notion of a more distributed organization of cognitive functions in women is supported by functional imaging studies revealing sex differences in the lateralization of language networks [46], with stronger bilateral processing in women than in men [47, 48].

Besides cortical lesions, subcortical strokes can cause apraxic deficits [49]. To date, only a few studies have investigated structural dysconnectivity in apraxia, applying different methods and tasks [50, 51, 52, 53]. The approach of disconnectome lesion–symptom mapping is promising for understanding the effect of subcortical lesions and white matter disconnection on apraxic deficits. The current findings on sex‐specific cortical lesion sites in apraxic patients with left‐hemisphere stroke hopefully trigger future studies to investigate sex‐related effects on dysconnectivity in apraxia.

### 4.4 Limitations

Even though there were no significant differences in the KAS scores reflecting apraxic deficits, we cannot rule out subtle differences in the praxis performance between men and women that are not reflected in the scoring scheme.

As we only included patients with unilateral LH stroke, we cannot make any inferences on sex‐related differences in the praxis networks of the right hemisphere. The data reported here stem from patients in the (sub)acute phase after stroke. Notably, regarding the longitudinal course, lesion sites associated with (persisting) apraxia in the chronic phase differ from those in the acute phase [54]. Individual recovery after stroke is influenced by numerous factors that may diverge between men and women and could modulate sex differences during rehabilitation and at the chronic stage of stroke.

While we controlled for many potential confounding factors, for example, age, handedness, aphasia severity, lesion location and size, stroke etiology, and time post‐stroke, some methodological caveats remain, such as the lack of information regarding socioeconomic status and ethnicity, and missing data on the education levels for one‐third of the patients, which may limit the generalizability of our results [55]. Notably, in the available patient data, there was no significant difference in education level between men and women. Furthermore, considering sex as a binary biological variable (as assigned at birth, indicating the gamete type) does not do justice to the complexity of sex variability [56]. For propositions on how to address and measure sex to advance future research in this field, please see [56] and note the ALBA guidelines for designing inclusive forms for gender and sexual diversity (https://www.alba.network/GSDinclusiveforms).

## 5 Conclusion

Our findings that apraxic deficits after LH stroke did not differ between men and women at the behavioral level but at the neural level, with a differential involvement of the left IFG in male compared to female LH stroke patients with apraxia, suggest a differential organization of the praxis network in men and women. Further studies on sex‐related differences in the praxis system are warranted to confirm and extend these findings.

## Author Contributions

**Nina N. Kleineberg:** conceptualization, investigation, writing – original draft, methodology, visualization, formal analysis, data curation, validation, software. **Claudia C. Schmidt:** writing – review and editing, methodology, conceptualization, data curation, validation, software. **Gereon R. Fink:** supervision, funding acquisition, writing – review and editing, project administration, resources, conceptualization. **Peter H. Weiss:** methodology, validation, writing – review and editing, supervision, resources, project administration, conceptualization.

## Conflicts of Interest

The authors declare no conflicts of interest.

## Supporting information


Data S1.

## Data Availability

The data that support the findings of this study are available on request from the corresponding author. The data are not publicly available due to privacy or ethical restrictions.

## References

[1] R. Cubelli , “Definition: Apraxia,” Cortex 93 (2017): 227, 10.1016/j.cortex.2017.03.012. 28410624

[2] T. E. Branyan and F. Sohrabji , “Sex Differences in Stroke Co‐Morbidities,” Experimental Neurology 332 (2020): 113384, 10.1016/j.expneurol.2020.113384. 32585156 PMC7418167

[3] A. K. Bonkhoff , G. Coughlan , V. Perosa , et al., “Sex Differences in Age‐Associated Neurological Diseases‐A Roadmap for Reliable and High‐Yield Research,” Science Advances 11, no. 10 (2025): eadt9243, 10.1126/sciadv.adt9243. 40043111 PMC11881909

[4] M. A. Heiskanen , J. Nevalainen , K. Pahkala , et al., “Change in Cognitive Performance During Seven‐Year Follow‐Up in Midlife Is Associated With Sex, Age, and Education – The Cardiovascular Risk in Young Finns Study,” Journal of Neurology 271 (2024): 5165–5176, 10.1007/s00415-024-12466-2. 38824491 PMC11319598

[5] K. Rodrigues Cavalcante and P. Caramelli , “Evaluation of the Performance of Normal Elderly in a Limb Praxis Protocol: Influence of Age, Gender, and Education,” Journal of the International Neuropsychological Society 15, no. 4 (2009): 618–622, 10.1017/s1355617709090663. 19573281

[6] F. Mauvais‐Jarvis , N. Bairey Merz , P. J. Barnes , et al., “Sex and Gender: Modifiers of Health, Disease, and Medicine,” Lancet (London, England) 396, no. 10250 (2020): 565–582, 10.1016/s0140-6736(20)31561-0. 32828189 PMC7440877

[7] L. Miola , V. Muffato , F. Pazzaglia , and C. Meneghetti , “Men's and Women's Egocentric and Allocentric Knowledge: The Involvement of Mental Rotation Ability and Spatial Beliefs,” Frontiers in Psychology 14 (2023): 1130549, 10.3389/fpsyg.2023.1130549. 36910832 PMC9995643

[8] D. Voyer , S. Voyer , and M. P. Bryden , “Magnitude of Sex Differences in Spatial Abilities: A Meta‐Analysis and Consideration of Critical Variables,” Psychological Bulletin 117, no. 2 (1995): 250–270, 10.1037/0033-2909.117.2.250. 7724690

[9] V. Kljajevic , H. R. Evensmoen , D. Sokołowski , J. Pani , T. I. Hansen , and A. K. Håberg , “Female Advantage in Verbal Learning Revisited: A HUNT Study,” Memory (Hove, England) 31, no. 6 (2023): 831–849, 10.1080/09658211.2023.2203431. 37114402

[10] L. Cartier , M. Guérin , F. Saulnier , et al., “Sex and Gender Correlates of Sexually Polymorphic Cognition,” Biology of Sex Differences 15, no. 1 (2024): 3, 10.1186/s13293-023-00579-8. 38191503 PMC10773055

[11] A. M. Proverbio , L. Ornaghi , and V. Gabaro , “How Face Blurring Affects Body Language Processing of Static Gestures in Women and Men,” Social Cognitive and Affective Neuroscience 13, no. 6 (2018): 590–603, 10.1093/scan/nsy033. 29767792 PMC6022678

[12] L. Sykes Tottenham , D. M. Saucier , L. J. Elias , and C. Gutwin , “Men Are More Accurate Than Women in Aiming at Targets in Both Near Space and Extrapersonal Space,” Perceptual and Motor Skills 101, no. 1 (2005): 3–12, 10.2466/pms.101.1.3-12. 16350603

[13] V. Wunderle , T. D. Kuzu , C. Tscherpel , G. R. Fink , C. Grefkes , and P. H. Weiss , “Age‐ and Sex‐Related Changes in Motor Functions: A Comprehensive Assessment and Component Analysis,” Frontiers in Aging Neuroscience 16 (2024): 1368052, 10.3389/fnagi.2024.1368052. 38813530 PMC11133706

[14] M. J. McKay , J. N. Baldwin , P. Ferreira , M. Simic , N. Vanicek , and J. Burns , “Normative Reference Values for Strength and Flexibility of 1,000 Children and Adults,” Neurology 88, no. 1 (2017): 36–43, 10.1212/wnl.0000000000003466. 27881628 PMC5200854

[15] M. Wallentin , “Putative Sex Differences in Verbal Abilities and Language Cortex: A Critical Review,” Brain and Language 108, no. 3 (2009): 175–183, 10.1016/j.bandl.2008.07.001. 18722007

[16] D. E. Aguilar Ramirez , J. Blinch , K. Robertson , J. Opdenaker , and C. L. R. Gonzalez , “Sex Differences in Visuospatial Cognition‐ a Female Advantage in Jigsaw Puzzle Solving,” Experimental Brain Research 242 (2024): 1821–1830, 10.1007/s00221-024-06845-4. 38847865

[17] L. Jäncke , “Sex/Gender Differences in Cognition, Neurophysiology, and Neuroanatomy,” F1000Research 7 (2018): 805, 10.12688/f1000research.13917.1. PMC6013760 29983911

[18] M. Hausmann , D. Schoofs , H. E. Rosenthal , and K. Jordan , “Interactive Effects of Sex Hormones and Gender Stereotypes on Cognitive Sex Differences—a Psychobiosocial Approach,” Psychoneuroendocrinology 34, no. 3 (2009): 389–401, 10.1016/j.psyneuen.2008.09.019. 18992993

[19] F. Sang , S. Zhao , Z. Li , Y. Yang , Y. Chen , and Z. Zhang , “Cortical Thickness Reveals Sex Differences in Verbal and Visuospatial Memory,” Cerebral Cortex 34, no. 3 (2024), 10.1093/cercor/bhae067. 38451300

[20] K. Zhang , H. Fang , Z. Li , T. Ren , B. M. Li , and C. Wang , “Sex Differences in Large‐Scale Brain Network Connectivity for Mental Rotation Performance,” NeuroImage 298 (2024): 120807, 10.1016/j.neuroimage.2024.120807. 39179012

[21] K. Chipman and E. Hampson , “A Female Advantage in the Imitation of Gestures by Preschool Children,” Developmental Neuropsychology 31, no. 2 (2007): 137–158, 10.1080/87565640701190692. 17488213

[22] K. Chipman and E. Hampson , “A Female Advantage in the Serial Production of Non‐Representational Learned Gestures,” Neuropsychologia 44, no. 12 (2006): 2315–2329, 10.1016/j.neuropsychologia.2006.05.002. 16780902

[23] K. G. Nicholson and D. Kimura , “Sex Differences for Speech and Manual Skill,” Perceptual and Motor Skills 82, no. 1 (1996): 3–13, 10.2466/pms.1996.82.1.3. 8668494

[24] A. Takasaki , M. Hashimoto , R. Fukuhara , et al., “Gesture Imitation Performance in Community‐Dwelling Older People: Assessment of a Gesture Imitation Task in the Screening and Diagnosis of Mild Cognitive Impairment and Dementia,” Psychogeriatrics: The Official Journal of the Japanese Psychogeriatric Society 24, no. 2 (2024): 404–414, 10.1111/psyg.13086. 38290836 PMC11577995

[25] J. Copley‐Mills , J. D. Connolly , and C. Cavina‐Pratesi , “Gender Differences in Non‐Standard Mapping Tasks: A Kinematic Study Using Pantomimed Reach‐To‐Grasp Actions,” Cortex 82 (2016): 244–254, 10.1016/j.cortex.2016.06.009. 27410715

[26] F. Gallo , A. González‐Villar , L. Ott , A. Sampaio , J. L. Nandrino , and A. Bartolo , “Gender Differences in the Observation of Gesture Direction: A Physiological Study,” Scientific Reports 14, no. 1 (2024): 23360, 10.1038/s41598-024-74082-4. 39375461 PMC11458844

[27] D. Kimura , “Sex Differences in Cerebral Organization for Speech and Praxic Functions,” Canadian Journal of Psychology 37, no. 1 (1983): 19–35, 10.1037/h0080696. 6196100

[28] Y. Silva , L. Sánchez‐Cirera , M. Terceño , et al., “Sex and Gender Differences in Acute Stroke Care: Metrics, Access to Treatment and Outcome. A Territorial Analysis of the Stroke Code System of Catalonia,” European Stroke Journal 8, no. 2 (2023): 557–565, 10.1177/23969873231156260. 37231687 PMC10334164

[29] P. Weiss , E. Kalbe , J. Kessler , et al., Das Kölner Apraxie Screening (Hogrefe Verlag, 2013).

[30] N. N. Kleineberg , C. C. Schmidt , M. K. Richter , et al., “Gesture Meaning Modulates the Neural Correlates of Effector‐Specific Imitation Deficits in Left Hemisphere Stroke,” NeuroImage: Clinical 37 (2023): 103331, 10.1016/j.nicl.2023.103331. 36716655 PMC9900453

[31] R. C. Oldfield , “The Assessment and Analysis of Handedness: The Edinburgh Inventory,” Neuropsychologia 9, no. 1 (1971): 97–113, 10.1016/0028-3932(71)90067-4. 5146491

[32] E. Kalbe , N. Reinhold , M. Brand , and J. Kessler , “The Short Aphasia‐Check‐List: An Economical Screening for Detecting Aphasia,” European Journal of Neurology 9 (2002): 209–210.

[33] E. Bates , S. M. Wilson , A. P. Saygin , et al., “Voxel‐Based Lesion‐Symptom Mapping,” Nature Neuroscience 6, no. 5 (2003): 448–450, 10.1038/nn1050. 12704393

[34] C. Rorden , H. O. Karnath , and L. Bonilha , “Improving Lesion‐Symptom Mapping,” Journal of Cognitive Neuroscience 19, no. 7 (2007): 1081–1088, 10.1162/jocn.2007.19.7.1081. 17583985

[35] C. C. Schmidt , E. I. S. Achilles , G. R. Fink , and P. H. Weiss , “Distinct Cognitive Components and Their Neural Substrates Underlying Praxis and Language Deficits Following Left Hemisphere Stroke,” Cortex 146 (2022): 200–215, 10.1016/j.cortex.2021.11.004. 34896806

[36] L. J. Buxbaum and J. Randerath , “Limb Apraxia and the Left Parietal Lobe,” Handbook of Clinical Neurology 151 (2018): 349–363, 10.1016/b978-0-444-63622-5.00017-6. 29519468 PMC8139361

[37] M. Metaireau , F. Osiurak , A. Seye , and M. Lesourd , “The Neural Correlates of Limb Apraxia: An Anatomical Likelihood Estimation Meta‐Analysis of Lesion‐Symptom Mapping Studies in Brain‐Damaged Patients,” Neuroscience and Biobehavioral Reviews 162 (2024): 105720, 10.1016/j.neubiorev.2024.105720. 38754714

[38] G. Goldenberg , J. Hermsdörfer , R. Glindemann , C. Rorden , and H. O. Karnath , “Pantomime of Tool Use Depends on Integrity of Left Inferior Frontal Cortex,” Cerebral Cortex 17, no. 12 (2007): 2769–2776, 10.1093/cercor/bhm004. 17339607

[39] L. J. Buxbaum , A. D. Shapiro , and H. B. Coslett , “Critical Brain Regions for Tool‐Related and Imitative Actions: A Componential Analysis,” Brain 137, no. Pt 7 (2014): 1971–1985, 10.1093/brain/awu111. 24776969 PMC4065019

[40] P. H. Weiss , S. D. Ubben , S. Kaesberg , et al., “Where Language Meets Meaningful Action: A Combined Behavior and Lesion Analysis of Aphasia and Apraxia,” Brain Structure and Function 221 (2016): 563–576. 25352157 10.1007/s00429-014-0925-3

[41] D. Y. Kimberg , H. B. Coslett , and M. F. Schwartz , “Power in Voxel‐Based Lesion‐Symptom Mapping,” Journal of Cognitive Neuroscience 19, no. 7 (2007): 1067–1080, 10.1162/jocn.2007.19.7.1067. 17583984

[42] G. Kim , E. Vitti , M. D. Stockbridge , J. L. Saver , A. E. Hillis , and A. V. Faria , “Association of Inferior Division MCA Stroke Location With Populations With Atrial Fibrillation Incidence,” Heliyon 9, no. 4 (2023): e15287, 10.1016/j.heliyon.2023.e15287. 37089357 PMC10113841

[43] A. L. Manuel , N. Radman , D. Mesot , et al., “Inter‐ and Intrahemispheric Dissociations in Ideomotor Apraxia: A Large‐Scale Lesion‐Symptom Mapping Study in Subacute Brain‐Damaged Patients,” Cerebral Cortex 23, no. 12 (2013): 2781–2789, 10.1093/cercor/bhs280. 22989580

[44] S. Caspers , K. Zilles , A. R. Laird , and S. B. Eickhoff , “ALE Meta‐Analysis of Action Observation and Imitation in the Human Brain,” NeuroImage 50, no. 3 (2010): 1148–1167, 10.1016/j.neuroimage.2009.12.112. 20056149 PMC4981639

[45] M. Heiser , M. Iacoboni , F. Maeda , J. Marcus , and J. C. Mazziotta , “The Essential Role of Broca's Area in Imitation,” European Journal of Neuroscience 17, no. 5 (2003): 1123–1128, 10.1046/j.1460-9568.2003.02530.x. 12653990

[46] M. Xu , X. Liang , J. Ou , H. Li , Y. J. Luo , and L. H. Tan , “Sex Differences in Functional Brain Networks for Language,” Cerebral Cortex 30, no. 3 (2020): 1528–1537, 10.1093/cercor/bhz184. 31512720

[47] L. C. Baxter , A. J. Saykin , L. A. Flashman , et al., “Sex Differences in Semantic Language Processing: A Functional MRI Study,” Brain and Language 84, no. 2 (2003): 264–272, 10.1016/s0093-934x(02)00549-7. 12590915

[48] B. A. Shaywitz , S. E. Shaywitz , K. R. Pugh , et al., “Sex Differences in the Functional Organization of the Brain for Language,” Nature 373, no. 6515 (1995): 607–609, 10.1038/373607a0. 7854416

[49] C. C. Schmidt , E. I. S. Achilles , K. Bolte , et al., “Association of Circumscribed Subcortical Gray and White Matter Lesions With Apraxic Deficits in Patients With Left Hemisphere Stroke,” Neurology 101, no. 11 (2023): e1137–e1144, 10.1212/wnl.0000000000207598. 37463748 PMC10513893

[50] F. E. Garcea , C. Greene , S. T. Grafton , and L. J. Buxbaum , “Structural Disconnection of the Tool Use Network After Left Hemisphere Stroke Predicts Limb Apraxia Severity,” Cerebral Cortex Communications 1, no. 1 (2020): tgaa035, 10.1093/texcom/tgaa035. 33134927 PMC7573742

[51] R. Metzgar , H. Stoll , S. T. Grafton , L. J. Buxbaum , and F. E. Garcea , “Single‐Case Disconnectome Lesion‐Symptom Mapping: Identifying Two Subtypes of Limb Apraxia,” Neuropsychologia 170 (2022): 108210, 10.1016/j.neuropsychologia.2022.108210. 35283160 PMC9189785

[52] H. Rosenzopf , D. Wiesen , A. Basilakos , et al., “Mapping the Human Praxis Network: An Investigation of White Matter Disconnection in Limb Apraxia of Gesture Production,” Brain Communications 4, no. 1 (2022): fcac004, 10.1093/braincomms/fcac004. 35169709 PMC8833454

[53] E. Rounis , E. Thompson , M. Scandola , et al., “A Preliminary Study of White Matter Disconnections Underlying Deficits in Praxis in Left Hemisphere Stroke Patients,” Brain Structure & Function 229 (2024): 2255–2268, 10.1007/s00429-024-02814-3. 39014269 PMC11611995

[54] A. Dressing , C. P. Kaller , M. Martin , et al., “Anatomical Correlates of Recovery in Apraxia: A Longitudinal Lesion‐Mapping Study in Stroke Patients,” Cortex 142 (2021): 104–121, 10.1016/j.cortex.2021.06.001. 34265734

[55] S. Bölte , J. Neufeld , P. B. Marschik , Z. J. Williams , L. Gallagher , and M. C. Lai , “Sex and Gender in Neurodevelopmental Conditions,” Nature Reviews Neurology 19, no. 3 (2023): 136–159, 10.1038/s41582-023-00774-6. 36747038 PMC10154737

[56] K. O. Smiley , K. M. Munley , K. Aghi , et al., “Sex Diversity in the 21st Century: Concepts, Frameworks, and Approaches for the Future of Neuroendocrinology,” Hormones and Behavior 157 (2024): 105445, 10.1016/j.yhbeh.2023.105445. 37979209 PMC10842816

