# Supplementary material for: Differential Lesion Patterns Associated With Stroke‐Induced Apraxia in Women and Men
Source: Eur J Neurol. 2025 May 23;32(5):e70201. doi: 10.1111/ene.70201 (PMC12100760; doi:10.1111/ene.70201)
Supplement: Supplementary file 1 — Data S1. [file ENE-32-e70201-s001.docx]

**Supplementary material to the manuscript “Differential lesion patterns associated with stroke-induced apraxia in women and men”**

**Suppl. section A:**

**Healthy control data**

**Methods**

We accessed data from 801 healthy controls, who had conducted the KAS (primarily in the framework of the *CRC 1451 on motor control*). These healthy participants had given written informed consent during their participation in the original studies of CRC 1451 that all their data may be used for further analyses and studies*.* Since all stroke patients were right-handed,

we did not consider participants that were left-handed according to the Edinburgh Handedness Inventory, i.e., with a laterality quotient of <40 (n=71), resulting in 426 healthy female and 303 healthy male participants. Using this sample, we conducted a case-control matching for sex and age with a tolerance of five years and group (patients/ healthy) as matching indicator. Thus, the matching was performed in accordance to the matching criteria that we had used to extract the age-matched sub-cohort of stroke patients with equal sex distribution from the initial cohort of apraxic stroke patients (n=102). For this patient sub-cohort (n=60), we extracted 60 sex- and age-matched healthy participants: 30 healthy female participants with an age of 66.5 ± 12.8 years (mean and standard deviation) and 30 healthy male participants with an age of 67.6 ± 11.6 years.

We then compared the KAS performance between healthy male and female participants accordingly (as we had done between male and female patients). Thus, in the healthy control cohort, we conducted a repeated measures ANOVA with the within-subject factors DOMAIN (pantomime vs. imitation) and EFFECTOR (bucco-facial vs. arm/hand) using SEX as between-subject factor. Further, the scores for the domains and effectors of the KAS were directly compared between men and women by Mann-Whitney-U test.

To relate the patient’s KAS performance to the healthy control data, we z-scored the individual patient scores to the respective sex group of the healthy controls, i.e.: individual’s z-scored KAS of a male patient = (KAS score of this individual male patient - mean KAS score of the healthy male group) / standard deviation of the KAS score of the healthy male group. Analogously, the z-scores for females and the respective subtests of the KAS were calculated.

**Results**

The 2x2 rm-ANOVA on the KAS scores revealed no significant main effect of SEX [F_(1,58)_=0.60, p=0.442] in the healthy participants. Furthermore, the two-way interactions DOMAIN x SEX [F_(1,58)_=1.62, p=0.208] and EFFECTOR x SEX [F_(1,58)_=3.05, p=0.086] and the three-way interaction of DOMAIN x EFFECTOR x SEX [F_(1,58)_<0.01, p=0.964] were not significant in the healthy participants. The scores for the domains and effectors of the KAS are shown in Suppl. Table S1. There was no significant difference in the performance between healthy males and females in this cohort age-matched to the patients.

The z-scored data relative to the healthy controls by sex also showed no significant differences in apraxic deficits between female and male LH stroke patients with apraxia. These analyses are essential for the interpretation of the patients’ sex specific results of the study.

**Suppl. Table S1:** Comparison of the matched healthy men and women in KAS performance

|  | **Healthy Females**  **N = 30** | **Healthy Males**  **N = 30** | **p-value** |
| --- | --- | --- | --- |
| **KAS total sum** | 75.7 ± 3.5,  76 [6], 66 - 80 | 75.0 ± 4.2,  75 [5], 59 - 80 | 0.513 |
| **KAS pantomime**  **subtests** | 36.5 ± 3.4,  37 [4.5], 26 - 40 | 36.7 ± 2.9,  37 [4.3], 27 - 40 | 0.988 |
| **KAS imitation**  **subtests** | 39.7 ± 1.5,  40 [2], 36 - 40 | 38.3 ± 2.1,  38 [2.5], 32 - 40 | 0.054 |
| **KAS bucco-facial**  **subtests** | 38.2 ± 1.8,  38 [3], 32 - 40 | 37.1 ± 3.0,  38 [4.3], 29 - 40 | 0.301 |
| **KAS arm/hand**  **subtests** | 37.6 ± 2.4,  38 [4], 33 - 40 | 37.9 ± 2.4,  38 [4], 30 - 40 | 0.527 |
| Values are shown as mean ± standard deviation, median [interquartile range], min - max. P-values are derived from the Mann-Whitney-U test | | | |

**Suppl. Table S2:** Patients KAS performance z-scored relative to the healthy controls by the respective sex

|  | **Female patients**  **N = 30** | **Male patients**  **N = 30** | **p-value** |
| --- | --- | --- | --- |
| ***KAS total sum*** | -4.0 ± 4.4,  -2.8 [4.0],  -19.4, 0.1 | -4.0 ± 4.5,  -3.1 [4.5],  -18.0, 0.3 | 0.906 |
| **KAS pantomime subtests** | -1.8 ± 2.9,  -0.7 [2.4],  -10.7, 1.0 | -2.3 ± 3.7,  -1.6 [5],  -12.7, 1.1 | 0.213 |
| **KAS imitation**  **subtests** | -5.7 ± 5.0,  -3.8 [5.3],  -21.3, 0.2 | -4.6 ± 4.7,  -3.0 [4.8],  -18.2, 0.8 | 0.224 |
| **KAS bucco-facial subtests** | -2.6 ± 4.2,  -1.2 [3.6],  -19, 1.0 | -1.7 ± 3.4,  -0.5 [3.3],  -12.4, 1.0 | 0.224 |
| **KAS arm/hand subtests** | -3.9 ± 3.9,  -2.3 [5.2],  -14.0, 1.0 | -4.8 ± 4.2,  -4.3 [6.8],  -15.8, 0.9 | 0.214 |
| Values are shown as mean ± standard deviation, median [interquartile range], min, max. P-values are derived from the Mann-Whitney-U test | | | |

**Suppl. section B:**

**Exploratory analyses on the interrelation of lesion sites revealed by the sex-specific VLSM analysis with apraxic deficits**

**Methods**

Based on the specific lesion sites (regions of interest, ROI) revealed by the VLSM of male versus female apraxic stroke patients (VLSM-ROI), we screened the patients whether their lesions included these differential sex-specific lesion sites for apraxia (VLSM-ROI), precisely, whether there was an overlap of 20 or more voxels between the individual stroke lesion and the VLSM-ROI. The cut-off value of 20 voxels was chosen according to the reporting threshold of significant clusters of our manuscript.

This resulted in i) a group of patients whose stroke lesions comprised the VLSM-ROI (mainly located in the IFG), and ii) a group of patients whose stroke lesions lay outside this VLSM-ROI. As an exploratory analysis assessing the influence of the VLSM-ROI on praxis performance, we performed a t-test between these two patient groups with the KAS score as the dependent variable.

**Results**

The exploratory analyses between the LH stroke patients whose stroke lesions comprised the differential sex-specific lesion sites for apraxia (VLSM-ROI, mainly located in the IFG, n=18) and the LH stroke patients whose stroke lesions lay outside this VLSM-ROI (n=42) revealed a significant effect on the KAS score [t(58)=2.03, p=0.047. LH stroke patients whose lesions involved the VLSM-ROI scored on average 9.6 points less in the KAS than patients whose lesion did not encroach onto the VLSM-ROI (53.4 ± 22.4 points versus 63.0 ± 22.4 points). Thus, LH stroke patients with lesions in the VSLM-ROI suffered from more severe apraxia. When considering the male LH stroke patients only, the KAS score difference was even 13.3 points (males with versus without lesions in the VLSM-ROI: 51.9 ± 23.8 versus 65.2 ± 8.2 points), while there was no significant difference in female patients (females with versus without lesions in the VLSM-ROI: 61.3 ± 13.6 versus 61.7 ± 15.7 points).
